# Positively-charged plasmonic nanostructures for SERS sensing applications

**DOI:** 10.1039/d1ra07959j

**Published:** 2022-01-04

**Authors:** Mariacristina Turino, Nicolas Pazos-Perez, Luca Guerrini, Ramon A. Alvarez-Puebla

**Affiliations:** Department of Physical and Inorganic Chemistry – EMaS, Universitat Rovira I Virgili Carrer de Marcel·lí Domingo s/n 43007 Tarragona Spain luca.guerrini@urv.cat ramon.alvarez@urv.cat; ICREA Passeig Lluís Companys 23 08010 Barcelona Spain

## Abstract

Surface-enhanced Raman (SERS) spectroscopy has been establishing itself as an ultrasensitive analytical technique with a cross-disciplinary range of applications, which scientific growth is triggered by the continuous improvement in the design of advanced plasmonic materials with enhanced multifunctional abilities and tailorable surface chemistry. In this regard, conventional synthetic procedures yield negatively-charged plasmonic materials which can hamper the adhesion of negatively-charged species. To tackle this issue, metallic surfaces have been modified *via* diverse procedures with a broad array of surface ligands to impart positive charges. Cationic amines have been preferred because of their ability to retain a positive zeta potential even at alkaline pH as well as due to their wide accessibility in terms of structural features and cost. In this review, we will describe and discuss the different approaches for generating positively-charged plasmonic platforms and their applications in SERS sensing.

## Abstract 2

Integration of ligands equipped with quaternary amines on plasmonic surfaces generates positively-charged nanomaterials suitable for electrostatically binding negatively-charged species paving the way for their application in SERS sensing.

## Graphical abstract

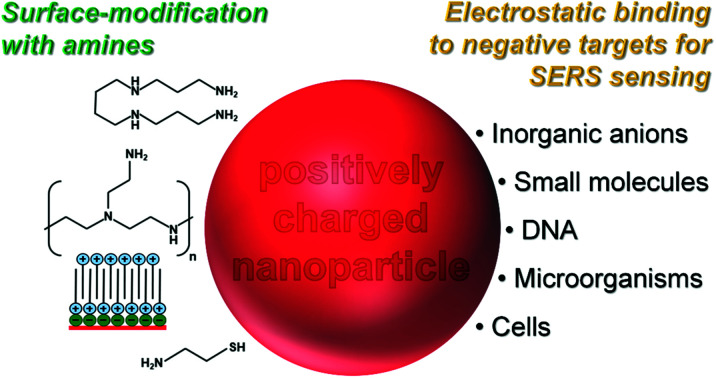

## 1. Introduction

Surface-enhanced Raman (SERS) spectroscopy is an ultrasensitive analytical tool that has been continuously expanding its range of applications in several fields, including biosensing, medicine, environmental monitoring, food safety, and catalysis.^1–10^ As the magnification of the Raman scattering primarily depends on the excitation of localized surface plasmon resonances (LSPRs) to generate intense electric fields at the metallic surfaces (mostly silver or gold), the design of the plasmonic substrate is central to SERS analysis. Besides the intrinsic optical response of the nanoconstruct, the fine-tuning of its surface physicochemical properties is key to maximizing the sensing performances as well as to guiding, when desired, its potential assembly into more complex architectures.^12–14^

Broadly speaking, SERS sensing strategies are generally classified based on two general principles: direct and indirect strategies. The direct methodologies rely on the acquisition of the intrinsic SERS spectra of the target species to detect its presence and, possibly, quantify its content in the sample.^5^ Conversely, indirect methods monitor the alteration of the optical signal originated from extrinsic SERS-active molecules whose extent, based on the appropriate design of the sensing platform, can be selectively and quantitatively correlated with the presence of the analyte. Notably, the most common implementation of indirect SERS is by using SERS encoded nanoparticles (also referred to as SERS tags or probes) which function similarly as fluorescent labels but with enhanced multiplexing, photostability, and quantitative response.^16,17^ Regardless of the sensing scheme, target species or SERS labels and chemosensing elements have to be brought into close contact with the metallic surface to yield intense SERS signals due to the exponential decay of the electric field intensity with distance from the plasmonic material.^6^ For indirect sensing, SERS labels and chemosensors are typically chosen among those carrying a mercapto group (or, to a lesser extent, a dithiocarbamate group) to promote the covalent attachment onto the nanostructure *via* the formation of the strong metal–S bond. In this case, then, the surface chemistry is tailored *via* ligand exchange upon displacement of the surface molecules retained at the plasmonic structure after the synthesis which, thus, makes this approach feasible only when the analyte displays higher affinity for the metal as compared to the native ligands.^12^ On the other hand, non-covalent interactions commonly takes place through electrostatic binding of the target onto the charged layer adhering onto the metallic surface, such as the well-studied interaction between surface halide anions (Cl^−^, Br^−^, I^−^) and quaternary aryl- and alkyl-ammonium compounds.^20,21^ Obviously, the chemistry of the metallic surface is directly related to the original synthetic procedures which, by and large, typically result in a negatively-charged interface formed by ligands such as citrate (and its oxidation products) and halide anions that can be found on conventional gold and silver colloids.^14^ Thus, the adsorption of negatively-charged analytes, especially those unequipped of reactive moieties capable of forming strong covalent bonds with the metal surface, has been traditionally hindered by charge repulsion. To tackle this issue, several strategies have been continuously developing to impart positive charge to the nanomaterial surface, including designing novel synthetic methods to yield intrinsically positive plasmonic materials as well as post-synthetic treatments to retool the pre-formed metal interface with appropriate capping ligands. Regardless of the approach, positively-charged plasmonic nanomaterials were obtained using surface ligand exposing quaternary ammonium groups towards the metal–liquid interface, mostly because of their ability to retain a positive zeta potential even at alkaline pH as well as their wide accessibility in terms of structural features and cost.^22^

Herein, we intend to provide a systematic overview on the topic by presenting and discussing the current state-of-the-art of the different methodologies of generation of positively-charged SERS substrates and their implementation into sensing platforms for detection of a wide range of analytes of interest. These targets were, for the sake of discussion, arbitrarily classified according to their relative size: from inorganic ions to biologically and environmentally relevant small molecules, from large biomolecules as nucleic acids to pathogenic microorganisms and cells.

## 2. Synthesis of positively-charged nanomaterials

Synthesis of positively-charged nanoparticles typically relies on two main strategies: the direct reduction of the metal precursor in the presence of positive stabilizing molecules or a post functionalization of previously formed nanoparticles (NPs) with molecules carrying positive groups. Both approaches have their advantages and drawbacks. The direct synthesis is usually easier and faster as it is performed *via* one-pot approaches. However, the monodispersity of the so-produced nanostructures is often relatively low with the additional problem of a limited tunability of NP size and morphology. On the other hand, post functionalization methods allow to better control the size and shape of the NPs using well-known established protocols for nanoparticle fabrication but frequently require tedious and complicated cleaning steps which can also be the source of undesired colloidal aggregation. Regardless, the surface functional moieties used up to date to impart positive charge to the nanomaterials are amine ligands of very diverse structural features. For the sake of discussion, these amine ligands were arbitrarily classified into the following groups: polymers, cationic surfactants, short polyamines and aminothiolated compounds. In this section, the most used strategies to produce positive NPs will be revised according to the different type of amino surface elements used.

### 2.1 Polymers

In general, the most common and easy way to produce positive NPs is *via* initial synthesis of conventional negative NPs followed by the subsequent coating with positive polymers, as similarly reported for the well-known layer-by-layer technique. In this case, standard negative citrate NPs can be easily turned into positive NPs by simply adding a solution containing a positive polymer. The most commonly used ones are polyethyleneimine (PEI),^23^ polyallylamine (PAA),^24^ poly-l-lysine (PLL),^25^ polyarginine (PA),^26^ and chitosan.^27,28^ This approach can be extended also to non-citrate stabilized NPs that exhibit negative charge on their surface as, for example, Au nanotriangles stabilized with dioctyl sodium sulfosuccinate (AOT)^29^ or Au nanostars stabilized with polyvinylpyrrolidone (PVP).^26^ This approach is very easy and effective however such coating process retains, as an intermediate layer between the metallic surface and the external positively-charged shell, the original surfactants. This can pose major limitations on their applicability for surface-dependent techniques like SERS. In addition, this type of coatings can suffer from degradation with time due to polymer detachment. Therefore, great efforts have been performed to achieve the direct synthesis of homogeneous NPs coated and stabilized with these amine ligands. For instance, the direct synthesis of Ag NPs has been achieved using PEI as a stabilizer and ascorbic acid as the reducing agent. The resulting Ag NPs show an average size of around 8.2 nm and the UV-vis extinction spectrum shows a strong plasmon resonance band at 399 nm.^30^ In a different report, larger Ag NPs of *ca.* 14 ± 6 nm were produced by simply boiling a AgNO_3_ solution in the presence of PEI without ascorbic acid.^31^ Further increase in the size of PEI-coated Ag nanoparticles of around 33 nm, was achieved at room temperature under UV irradiation for 120 min in the presence of 4-(2-hydroxyethyl)-1-piperazineethanesulfonic acid (HEPES) and PEI.^32,33^

**Fig. 1:**
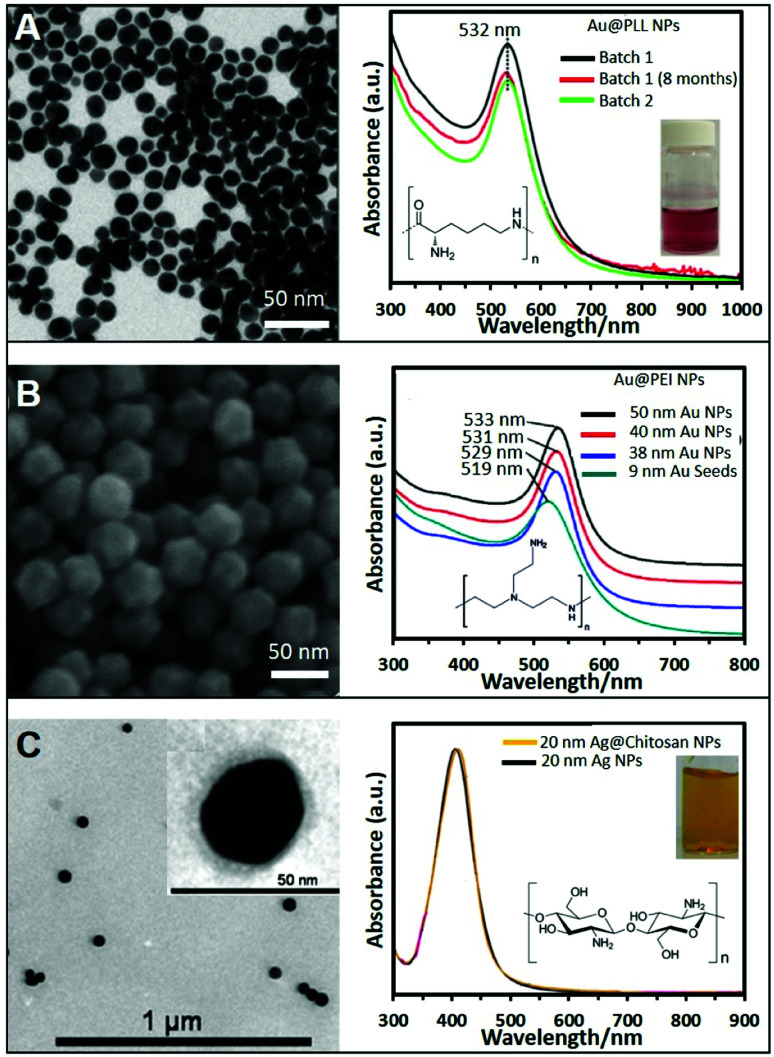
(A) TEM image of gold nanoparticles (Au NPs) stabilized with poly-l-lysine and the corresponding UV-vis spectra showing the reproducibility and stability of the produced NPs; the inset shows a photograph of the colloidal dispersion. Adapted with permission from ref. 34 Copyright 2020, Springer. (B) SEM image of 50 nm Au NPs stabilized with PEI and produced by the seeded growth method. UV-vis absorption spectra of PEI-coated NPs of different sizes (9, 38, 40, and 50 nm). Adapted with permission from ref. 36 Copyright 2018, Elsevier. (C) TEM image of 20 nm chitosan-coated silver nanoparticles (Ag NPs). The chitosan layer can be clearly distinghuised in the inset image. UV-vis spectra of Ag NPs in the presence of chitosan (orange) and without (black). Adapted with permission from ref. 38. Copyright 2011, IOP Publishing Ltd.

Besides PEI, other polymers have been successfully exploited in the direct synthesis of cationic NPs using the same one-pot strategy. For example, the highly biocompatible polypeptide poly-l-lysine (PLL) has been employed to produce Au@PLL the NPs showed in Fig. 1A by simply mixing the gold precursor with PLL while boiling. Fig. 1A also shows the UV-vis absorption spectrum of two different batches of Au@PLL NPs and after eight months. All of them have a LSPR peak at 532 nm without appreciable changes indicating the reproducibility of the synthetic procedure and the long-term stability.^34^ A similar approach has been used with chitosan, a polysaccharide bioactive polymer with a wide variety of applications due to its functional properties such as antibacterial activity, non-toxicity, ease of modification, and biodegradability. For instance, HAuCl_4_ and chitosan were combined and heated at ∼95 °C for ∼3 h to generate the corresponding chitosan-coated nanoparticles.^35^

While these approaches offer very straightforward routes to produce positive nanoparticles, they typically yield relatively small colloids with subpar SERS-enhancing performances. Therefore, great efforts have been devoted to the fabrication of larger and homogeneous plasmonic NPs *via*, among others, seed-mediated approaches. For instance, the direct synthesis of well-defined polyhedral gold nanoparticles with various sizes (38, 40, and 50 nm) using PEI as a capping molecule has been successfully achieved using preformed PEI-coated gold seeds of 9 nm. Size tuning was achieved by combining different amounts of seed to a growth solution containing HAuCl_4_ and PEI (ascorbic acid was employed as the reducing agent). Fig. 1B shows a SEM image of the 50 nm nanoparticles, demonstrating the monodispersity of the produced structures, as well as the red-shift of the LSPR maximum with the nanoparticle size (Fig. 1B).^36,37^

Another two-step growth method has been proposed to produce chitosan-coated Ag NPs. In the first step, a stock of negatively-charged silver seeds was synthesized *via* reduction of silver nitrate with sodium borohydride in the presence of sodium citrate. In a second step, an aqueous solution of trisodium citrate, ascorbic acid, chitosan, and seeds was prepared. To this mixture, AgNO_3_ was added dropwise under continuous magnetic stirring at 0 °C. Fig. 1C shows a TEM image of the so prepared Ag spherical particles with an average diameter of 20 nm. The inset shows a higher magnification TEM image of the stained NPs showing the presence of the thin chitosan outer layer. The optical spectrum of the chitosan-coated NPs shows a LSPR centered at around 410 nm, approximately 5 nm more than the chitosan-free colloids (Fig. 1C), which been associated to the change in the local refractive index of nanoparticles resulting from the polymer coating.^38^ Following these protocols, it is also possible to produce Ag/Au bimetallic NPs by first synthesizing Ag nanoparticles and then induce a controlled galvanic replacement reaction by the stepwise addition of HAuCl_4_ while boiling under constant stirring.^39^ In addition, cationic Ag layers have been also grown on colloidal templates like for instance poly(glycidyl methacrylate) (PGMA) nanoparticles. To this end, PGMA NPs were previously coated with PEI to modify the nanoparticle charge to further grow Ag nanoparticles on the surface by adding a AgNO_3_ solution and NaBH_4_.^40^

### 2.2 Cationic surfactants

**Fig. 2:**
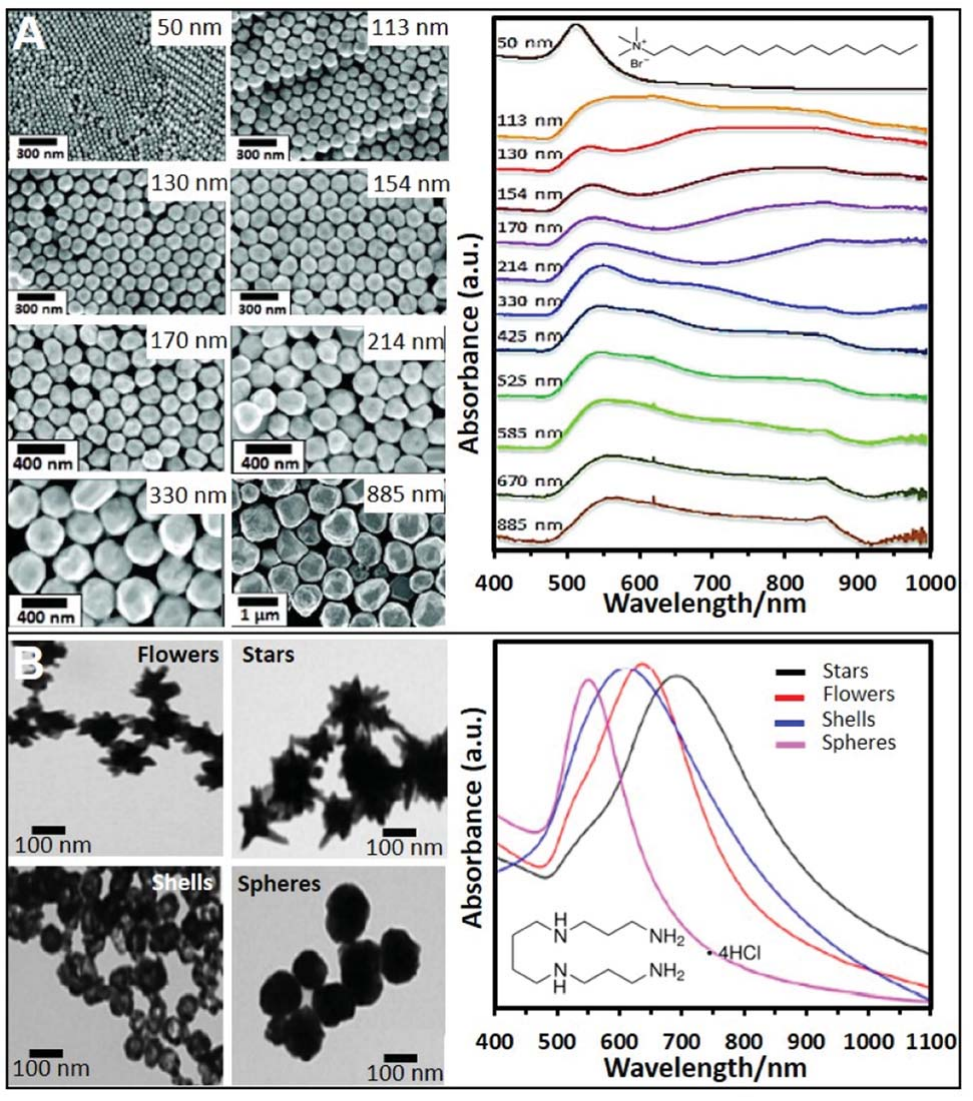
(A) SEM images of CTAB-stabilized Au NPs from 50 to 885 nm diameter size and the corresponding UV-vis spectra. Adapted with permission from ref. 41 Copyright 2012, American Chemical Society. (B) TEM images and corresponding UV-vis spectra of four different nanoparticle morphologies (flowers, stars, shells, and spheres) produced using a seedless and surfactant-free method and subsequently coated with spermine molecules. Adapted with permission from ref. 60. Copyright 2020, American Chemical Society.

Cationic surfactants like cetyltrimethylammonium bromide (CTAB), cetyltrimethylammonium chloride (CTAC), and, to a lesser extent, benzyldimethyldodecylammonium chloride (BDAC) are widely used to produce gold structures of different geometries and sizes. Seed mediated protocols using these surfactants allow the control over the size and morphology of the produced NPs with a high degree of tunability. For instance, spherical Au NPs from sizes ranging from 50 nm to 1 μm can be produced by preparing a seed solution by reducing HAuCl_4_ in the presence of sodium citrate with NaBH_4_. To synthesize Au NPs of different sizes, a specific amount of the as-prepared seeds was added to a growth solution containing CTAB, HAuCl_4_, potassium iodide, and ascorbic acid. Fig. 2A shows SEM images of the produced NPs and the trend corresponding extinction spectra. As it can be seen, 50 nm NPs show the characteristic dipolar plasmon mode around 532 nm. However, as particle size increases, the band is noticeably red-shifted and broadened. For NPs above *ca.* 113 nm, a shoulder appears at lower wavelengths which corresponds to the quadrupolar resonance. From particles sizes above 330 nm, scattering effects dominate the spectra.^41^ This synthetic protocol has been also reproduced replacing CTAB with CTAC, which avoids the use of iodide.^42^

CTAB, CTAC and BDAC were also employed in the production of anisotropic NPs such as gold nanorods (NRs),^21,43^ bipyramids,^44^ or nanostars (NSt).^45^ By preparing the seeds in the presence of these surfactants rather than citrate and replacing the iodide with AgNO_3_ to break the symmetry of the seeds, Au NRs can be obtained.^30,31,46^ The use of seeds formed in the presence of citrate and CTAC, and aged for 90 min at 80 °C, enabled the formation of bipyramids.^44^ The production of positive Ag NPs with tunable sizes is also possible using CTAB with a similar approach as for Au. The main difference is that a base like NH_4_OH has to be added to initiate the reduction process.^47–51^

Such a class of surfactants forms a bilayer around the NPs which imparts colloidal stability while providing a net positive surface charge. However, nanoparticle aggregation is prevented only for surfactant content above the critical micellar concentration (*i.e.*, when a large content of unbound surfactant molecules is still present in the solution).^44^ Furthermore, the surfactant bilayer can significantly hinder the ability of target molecules to approximate the metallic surface (the CTAB bilayer has been reported to be around 3–5 nm thick).^52^

### 2.3 Small molecules

Short polyamine molecules (*e.g.*, spermine; spermidine, and ethylenedioxydiethylamine) were also used to fabricate positively-charged plasmonic colloids. Among them, spermine (Sp) tetrahydrochloride has been the most widely employed chemical. Sp molecules adhere onto the NP surfaces *via* electrostatic binding and adopting a flat orientation, which ideally minimizes the separation between the plasmonic materials and the media. This type of NPs is generally prepared by adding NaBH_4_ to a solution containing AgNO_3_ and spermine. By performing this approach and adding different amounts of NaBH_4_ different sizes of Ag NPs can be produced.^53–56^ For instance, spermine coated Ag NPs of 20, 40, and 60 nm could be obtained. Their corresponding surface plasmon resonance bands showed a redshift with increasing Ag NP size, 394 nm, 400 nm, and 413 nm respectively.^57^ The corresponding zeta potentials reported in a different work for similar sizes were 13, 8.4, and 21 mV.^19^

Nonetheless, even when reported, the possibility to produce different NPs sizes using spermine remains elusive. Therefore, other strategies using preformed NPs have been also proposed. For instance, citrate-capped Ag colloids were cleaned by centrifugation and mixed with an equal volume of spermine solution, adopting a similar post-modification strategy as for polymer coatings.^58^ Using this approach, spermine-coated gold nanoparticles of different morphologies (nanostars, nanoflowers, or nanoshells, see Fig. 2B) were obtained.^59,60^

### 2.4 Aminothiolated compounds

Besides electrostatic anchoring of the surface ligands onto the metallic nanomaterials, formation of direct covalent bonds has been exploited by using amino molecules equipped with an addition thiol or, to a lesser extent, dithiocarbamate group.

Several approaches have been proposed for their direct synthesis. Gold nanoparticles have been produced by the dropwise addition of sodium borohydride to an aqueous solution of tetrachloroauric(iii) acid under magnetic stirring at room temperature. Once the solution became red, indicating the NPs formation, a solution of cysteamine hydrochloride was added as stabilizing agent.^61,62^ The same approach has been used to produce Ag NPs that were further stabilized by the addition of l-cysteine. In this case, sodium borohydride was added dropwise to a silver nitrate solution under stirring.^63^ Alternatively, positively-charged Au NPs were obtained by mixing first the thiolated molecule 2-aminoethanethiol with the metal precursor (HAuCl_4_) followed by the addition of NaBH_4_.^64^ However, nanoparticles obtained using NaBH_4_ as a reducing agent are typically small (*ca.* 12 nm). Larger NPs (*ca.* 51 nm) were then produced by replacing NaBH_4_ with heating (cysteamine was added to a boiling solution of HAuCl_4_ under magnetic stirring). However, in the reported protocol, thiolated polyethylene glycol (SH-mPEG) was added to improve the stability of the synthesized Au NPs.^65^

**Fig. 3:**
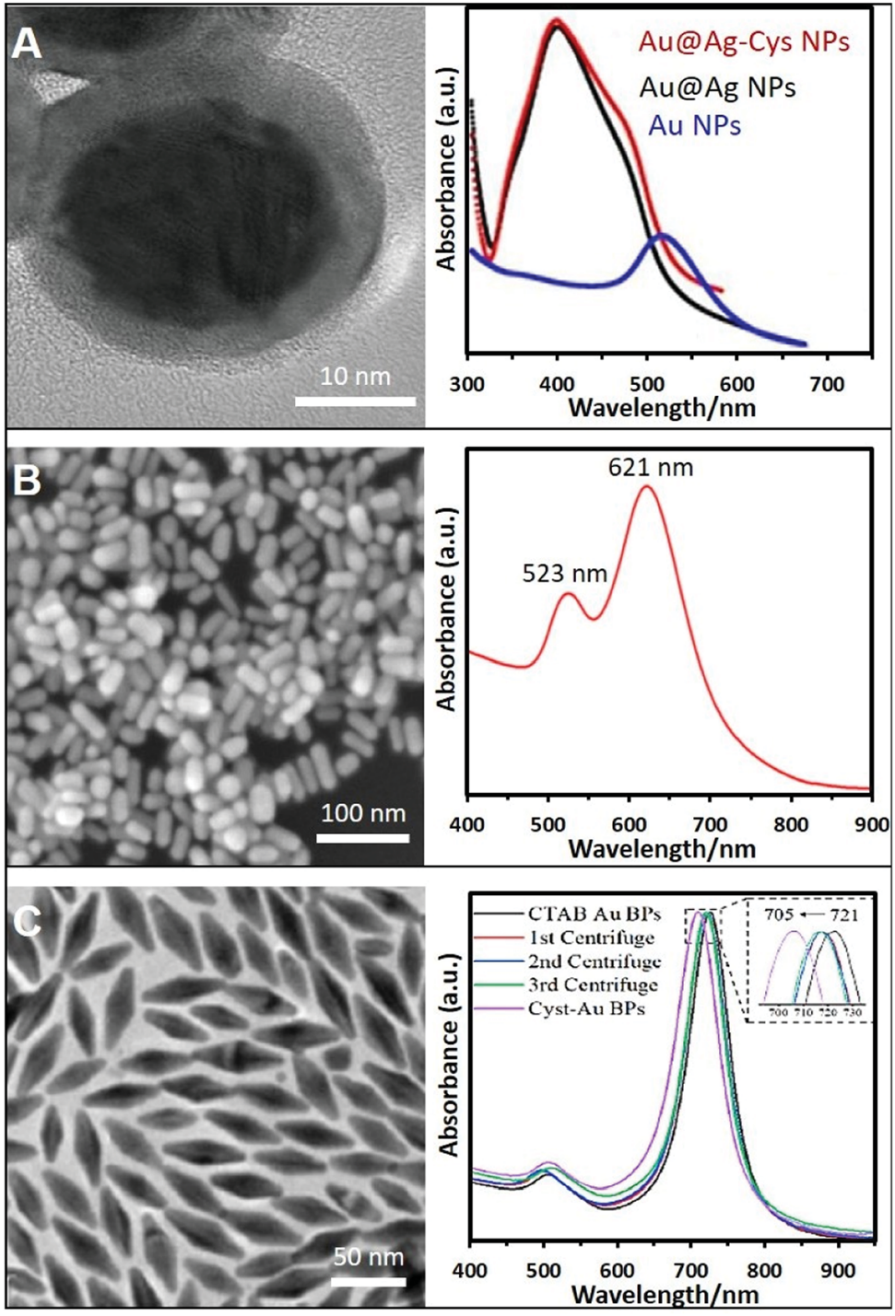
(A) High-resolution TEM image of Au@Ag NPs functionalized with cysteamine and their extinction spectrum (those of the AuNP core and non-modified Au@AgNPs were also included). Adapted with permission from ref. 69 Copyright 2020, Elsevier. (B) SEM image and extinction spectra of Au nanorods (NRs) functionalized with cysteamine. Adapted with permission from ref. 73 Copyright 2020, Elsevier. (C) TEM image of Au bipyramids and the monitoring of the ligand exchange process from CTAB to cysteamine *via* UV-vis spectroscopy. Adapted with permission from ref. 44 Copyright 2021, American Chemical Society.

Nevertheless, the control over the size and shape of the plasmonic nanoparticles remains very limited by the direct synthesis in the presence of thiols. Thus, the post-functionalization of preformed plasmonic nanoparticles with thio–amino molecules has been often reported to overcome this limitation. In this regard, citrate-capped nanoparticles were largely used as colloidal materials to generate positively-charged colloids by simple mixing, under constant agitation, with aminothiolated molecules such as cysteamine hydrochloride,^66–69^ diethyldithiocarbamate (DDTC),^70^ thiocholine,^22^ or l-cysteine methyl ester hydrochloride.^71^Fig. 3A shows an example where Ag was grown over preformed Au NPs and further functionalized with cysteamine. The same protocol with small modifications can be applied to NPs stabilized whit different surfactants or polymers like PVP or CTAB. For instance, PVP–Au NPs need to be centrifuged twice to remove excess PVP before functionalization with the thioamine solution (cysteamine).^72^ In the case of CTAB, CTAC, or BDAC stabilized NPs, the nanomaterials must be submitted to carefully tuned washing cycles to remove the excess of the surfactants while retaining the colloidal stability. Subsequently, the nanoparticles, such as Au NRs,^73^ or bipyramids,^44^ were combined with a cysteamine hydrochloride solution under magnetic stirring at 40 °C. The corresponding SEM and TEM images with their optical characterization are shown in Fig. 3B and C. In the same way, Au NSts prepared with a mixture of CTAB/BDAC were functionalized l-cysteine.^45^

In Table 1 the most common strategies for the direct synthesis of spherical positive plasmonic nanoparticles are summarized.

**Table 1:** Summary of the most common strategies to synthesize positively-charged plasmonic nanospheres

Stabilizing	Metal	Reductant	NP size (nm)
PEI^32,33^	Au	NaBH_4_	9
Spermine^57^	Ag	NaBH_4_	20–60
Cysteamine^61,62^	Au	NaBH_4_	12
l-Cysteine^63^	Ag	NaBH_4_	22
PEI^31^	Ag	*T* (90 °C)	14
Chitosan^35^	Au	*T* (95 °C)	17
Cysteamine^65^	Au	*T* (100 °C)	51
PEI^30^	Ag	Asc. Ac + *T* (90 °C)	8
PEI^28,29^	Ag	UV irradiation	33
PEI^32,33^	Au	Asc. Ac + seeds	37–100
Chitosan^38^	Ag	Citrate + Asc. Ac + seeds	20
CTAB/CTAC^41,42^	Au/Ag	Asc. Ac + seeds	10–100

To summarize, post-coating of pre-formed colloids with polymers such as PEI offers a straightforward route to obtain positive nanoparticles with resistance against aggregation. However, the relatively large thickness of the polymeric layer can limit the suitability of the substrates to SERS applications by acting as an efficient spacer from the plasmonic surface, as we will further discuss in the next section. Post-functionalizations with smaller-sized surface ligands such as short amino molecules (*e.g.*; spermine), which are electrostatically adhering onto the colloids similarly as the polymeric species, or covalently-bound amino-thiolated molecules, facilitate the closer contact of the plasmonic surface with the negatively-charged analyte. Although, it is frequently accompanied by a reduction of the colloidal stability. On the other hand, direct fabrication of positively-charged nanoparticles is typically limited to spherical nanoparticles with, often, relatively small diameters and broad size distributions, except for cationic surfactant-based synthesis (*e.g.*, CTAB). However, in the latter case, the residual surfactant bilayer at the plasmonic surface can, once again, limit the analyte accessibility to the nanomaterial while imparting colloidal stability only for relatively high surfactant concentration in the media (*i.e.*, above the critical micellar concentration). Since there is no ideal positively-charged nanomaterial to be used as universal SERS substrates, each specific application with its distinct requirements will determine the substrate of choice.

Finally, it is also worth stressing that the positively-charged nanomaterials can be integrated into hybrid composites comprising additional building units (*e.g.*, magnetic particles, inert supports, quantum dots, microfluidic chips, *etc.*) to generate multifunctional materials with synergistic properties. These types of multifunctional nanostructures are generally produced in multistep processes where the individual components are separately synthesized and subsequently assembled.^2,68,74,75^

## 3. SERS sensing applications

SERS-sensing with positively-charged nanostructures typically relies on electrostatic binding of the target species with the amino groups of the surface ligand to promote its adhesion onto the plasmonic surface while the detection is mostly achieved *via* the acquisition of the intrinsic SERS spectrum of the analyte (*i.e.*, direct SERS). However, we will also discuss indirect sensing strategies using positively-charged nanoparticles as SERS-encoded nanoparticles or SERS tags. For the sake of discussion, the studies reported in the literature were arbitrarily classified and discussed according to the size range of the target molecules.

### 3.1 Inorganic anions

**Fig. 4:**
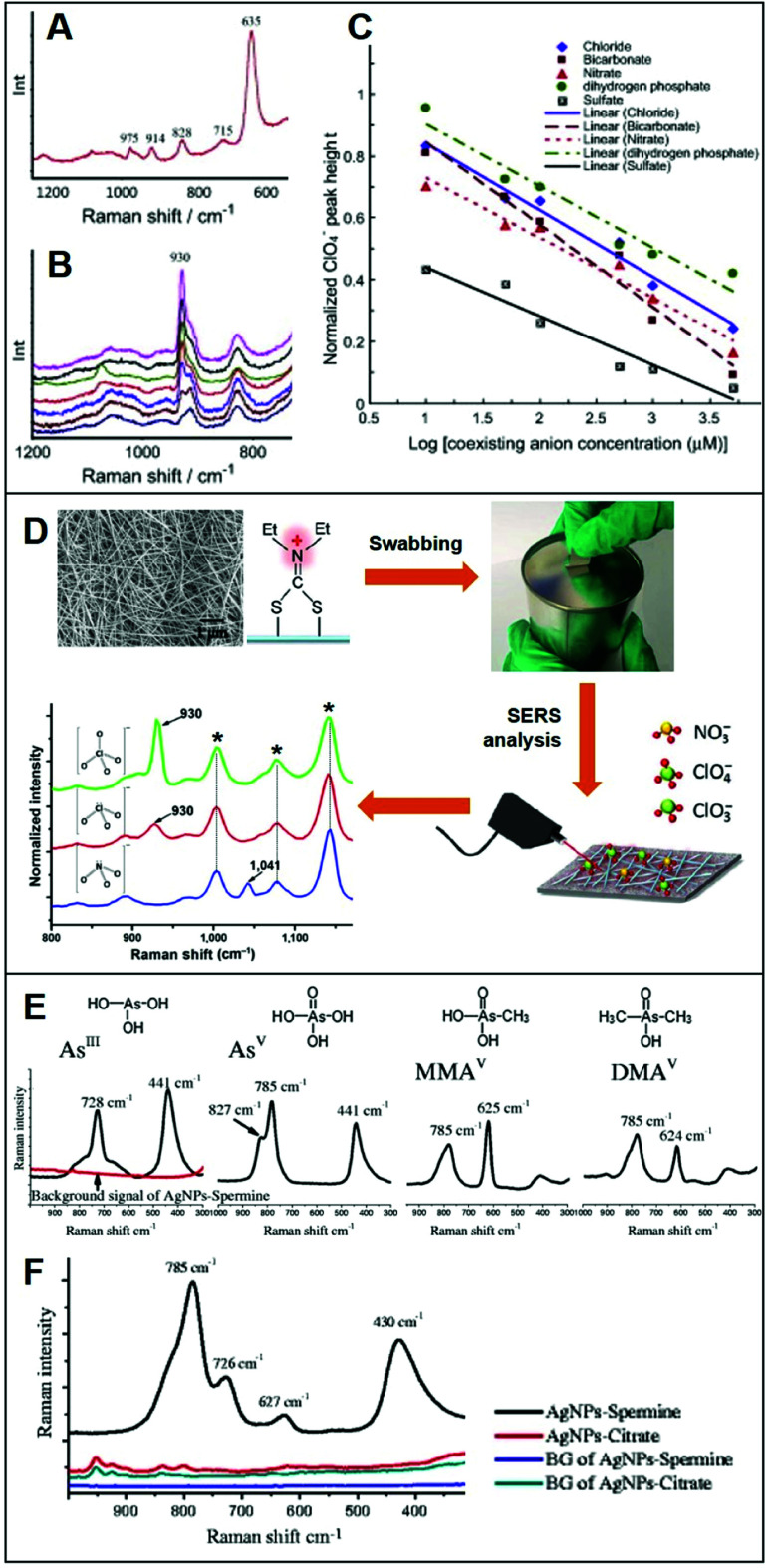
Direct SERS detection of inorganic anions. (A) Background spectrum of the Cys on Ag nanofilm/Cu foil in deionized water. (B) SERS spectra of a 50 μM ClO_4_^−^ solution in the presence of different concentrations of HCO_3_^−^ (0, 10, 50, 100, 500, 1000 and 5000 μM, from top to bottom). (C) Normalized ClO_4_^−^ peak height plotted against concentration of coexisting anions (Cl^−^, NO_3_^−^, SO_4_^2−^, HCO_3_^−^ and H_2_PO_4_^−^) in logarithmic scale (log μM). Adapted with permission from ref. 79 Copyright 2015, Elsevier. (D) SEM image of the Ag nanowire membrane and surface modification with diethyldithiocarbamate (DDTC) which is used as a swab to collect trace amounts of ClO_4_^−^, ClO_3_^−^ and NO_3_^−^ anions onto a surface. SERS analysis of the swabbed membrane onto surfaces with 2.0 μg of ClO_4_^−^, 1.7 μg of ClO_3_^−^ and 1.2 μg of NO_3_^−^ (asterisks: DDTC bands). Adapted with permission from ref. 80 Copyright 2016, Springer. (E) Individual SERS spectra of fours arsenic species (4 ppm) on spermine-coated silver nanoparticles (AgSp). (F) SERS spectra of a mixture of the four arsenic species (200 ppb) in the cell lysate on negatively-charged citrate silver colloids (AgCit) and AgSp. The background signals AgCit and AgSp were also included. Adapted with permission from ref. 56 Copyright 2017, Springer.

Integration of positively-charged plasmonic nanomaterials into SERS sensing schemes for the quantification of inorganic anions of human and environmental concern^22,32,33,56,70,76–80^ has been extensively investigated in the literature. For instance, Hao *et al.*^79^ fabricated a Ag nanofilm on Cu foils which, subsequently, was immersed into a cysteamine (Cys) solution to generate a Cys-self-assembled monolayer (SAM) for the direct SERS detection of perchlorate (ClO_4_^−^), a ubiquitous environmental contaminant. The SERS spectrum of Cys (Fig. 4A) displays, among others, an intense band at *ca.* 635 cm^−1^, assigned to the C–S stretching of Cys *gauche*-conformation and a weaker feature at *ca.* 715 cm^−1^ ascribed to the Cys *trans*-conformation, which suggests that the *gauche* conformer is predominant in the SAM. Immersion into a 50 μM perchlorate solution leads to the appearance of the characteristic ClO_4_^−^ band at *ca.* 930 cm^−1^ (Fig. 4B) which intensity decreases in the presence of an increasing concentration of co-existing anions, such as bicarbonate (Fig. 4B), indicating the following order of selectivity: ClO_4_^−^ > SO_4_^2−^ > HCO_3_^−^, NO_3_^−^ > Cl^−^ > H_2_PO_4_^−^ (Fig. 4C). Shi *et al.*^80^ fabricated a positively-charged silver nanowire membrane *via* post-functionalization of the PVP-capped wire with diethyldithiocarbamate (DDTC, Fig. 4D). The SERS-active, flexible membrane has been used to recover typical oxidizers contained in explosives (perchlorates, ClO_4_^−^; chlorates, ClO_3_^−^; and nitrates, NO_3_^−^) present at the nanogram levels on surfaces *via* simple surface swab. This enables the rapid on-site SERS detection using a portable Raman spectrometer *via* the collection of the characteristic bands of ClO_4_^−^/ClO_3_^−^ at 930 cm^−1^ and NO_3_^−^ at 1041 cm^−1^. Contrarily, unmodified-Ag nanowire membranes did not allow the detection of the target analytes.

On the other hand, Yang *et al.*^56^ exploited spermine-coated silver colloids (AgSp) to detect toxic arsenic species such as arsenite (As^III^), arsenate (As^V^), and two biologically relevant metabolic products of arsenic (monomethylarsonic acid, MMA^V^; and dimethylarsinic acid, DMA^V^) in cell lysates. Arsenic speciation in biological samples is important to better discern the impact of arsenic toxicity and its mode of action. Fig. 4D shows the SERS spectra of all arsenic species on AgSp colloids, together with the molecular structures of each analyte. For comparison, the authors also tested negatively-charged citrate silver colloids (AgCit) of similar size, showing that arsenic speciation in cell lysate can only be achieved on positively-charged colloids, while AgCit do not yield distinguishable SERS signals under the applied experimental conditions (Fig. 4E illustrates the SERS spectra obtained by a mixture of the arsenic species combined with the two colloids).

### 3.2 Small organic molecules

In addition to inorganic anions, a large set of small negatively-charged organic molecules such as explosives,^22,48,68,70,71,81–83^ pollutants,^28,66,73,84,85^ and pigments^25,31,44^ has been targeted by SERS-sensing approaches using positively-charged plasmonic nanomaterials.

**Fig. 5:**
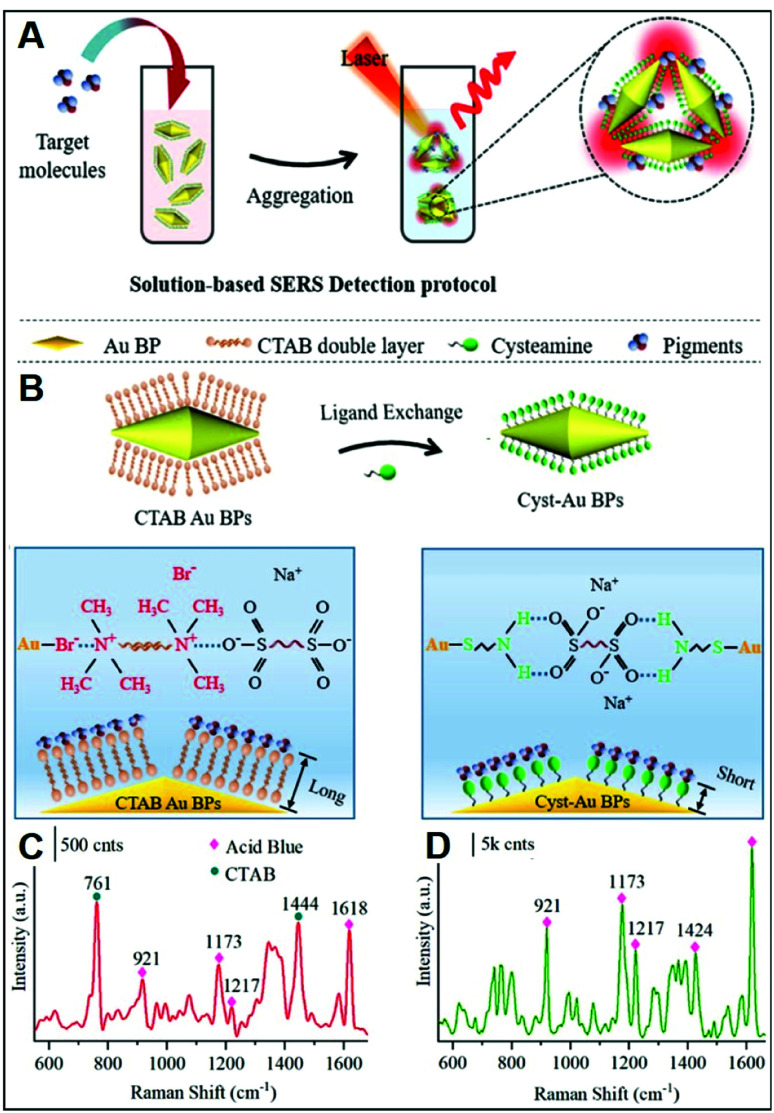
Direct SERS detection of pigments. (A) Outline of the sensing scheme for pigment detection using positively-charged gold nano-bipyramids (AuBPs). (B) Surface modification of CTAB-capped AuBPs with cysteamine. (C and D) SERS spectra of acid blue (1 ppm) using CTAB-capped (C) and cys-Au BPs (D) together with an illustration of the electrostatic interaction with their respective surface molecules. Adapted with permission from ref. 44 Copyright 2021, American Chemical Society.

As previously discussed, CTAB is a surfactant commonly used as a shape-directing agent to yield asymmetric nanoparticles with tunable and improved plasmonic responses as compared to the traditional spherical counterparts. While imparting an overall positive charge, the CTAB bilayer at the metallic surface also negatively impacts the ability of analytes to closely approach the plasmonic nanostructures and, thus, the possibility of acquiring intense SERS spectra of the molecular target. In this regard, an illustrative example is provided by the work of Amin *et al.*,^44^ who first synthesized CTAB-capped gold nano-bipyramids (BP–CTAB) and then submitted to ligand exchange with cysteamine (Cys) to generate the corresponding BP–Cys materials (Fig. 5A). The addition of negatively-charged pigments (*e.g.*, acid blue) to the colloidal suspensions causes the electrostatic-induced aggregation of the nanoparticles and the formation of interparticle hot-spots that boost the SERS signal (Fig. 5B). While characteristic acid blue bands are observed on both colloids, their intensities are much larger for BP–Cys (Fig. 5C and D) which is consistent with the thinner spacing layer determined by the self-assembling of small Cys molecules on the gold surface.

**Fig. 6:**
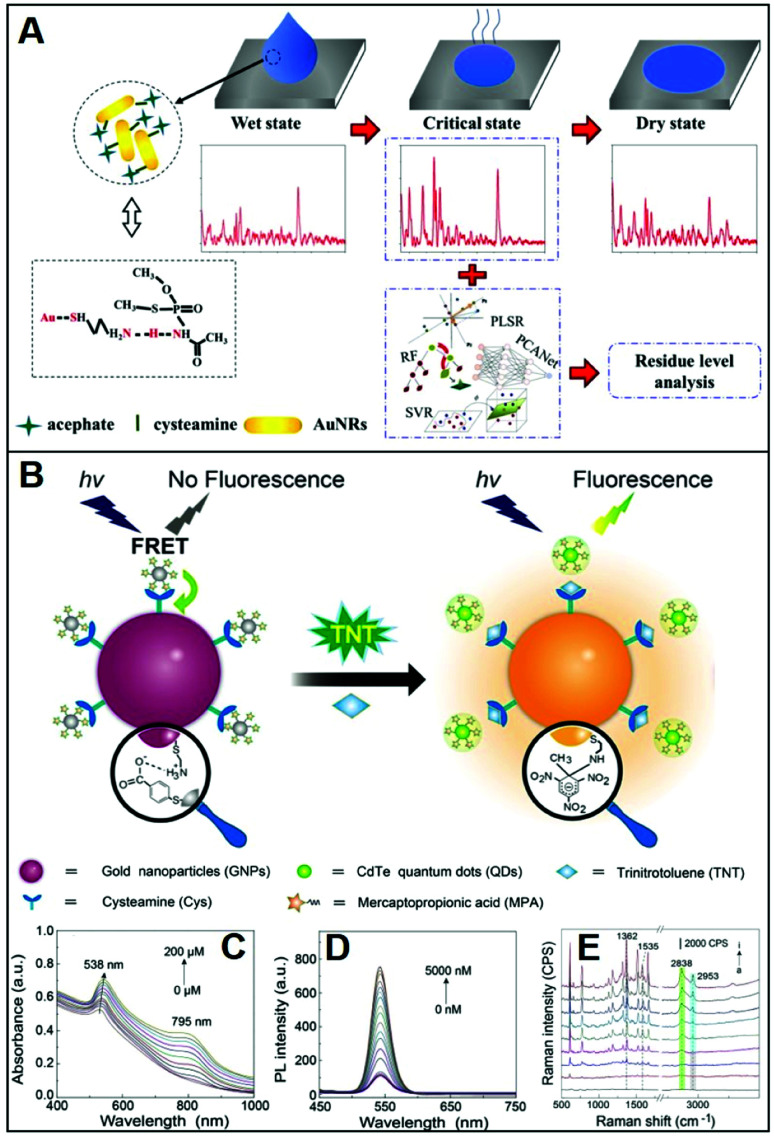
Direct SERS detection of small hazardous molecules. (A) Outline of the SERS detection of acephate in rice using dynamic SERS analysis in combination multivariant methods and cysteamine-modified gold nanorods as SERS substrates. Adapted with permission from ref. 73 Copyright 2020, Elsevier. (B) Depiction of the tri-modal sensing platform (colorimetric, fluorescence, and SERS) consisting of negatively-charged satellites (mercaptopropionic acid-capped CdTe quantum dots, QDs) assembled on a positively-charged plasmonic core (cysteamine-modified gold nanoparticles, GNP) for TNT analysis: extinction (C), photoluminescence (D) and SERS (E) spectra of GNPs-QDs assemblies in the presence of increasing concentrations of TNT (0–200 μM, 0–6000 nM and 0–100 nM, respectively). Adapted with permission from ref. 68 Copyright 2021, Elsevier.

Adopting an analogous approach, Weng *et al.*^73^ replaced the CTAB bilayer on gold nanorods (NRs) with Cys for the SERS detection of the toxic pesticide acephate in rice samples over a broad dynamic range of concentration and down to 0.5 mg L^−1^. In this case, however, NR–Cys colloids were combined with a solution extracted from acephate-contaminated rice and dropped on a silicon chip. SERS spectra were then continuously acquired during the drying process, which can be broadly separated into three distinct stages: wet state, critical state, and dry state (Fig. 6A). Maximum SERS intensities, for 785 nm excitation laser, are observed during the second stage, which has been attributed to the peculiar geometrical organization of the NR–Cys into a 3D long-range-ordered array that optimizes the generation of closely-spaced interparticle gaps. SERS spectra acquired during the critical state display, among others, characteristic features of acephate centered at *ca.* 358, 400, 560, and 692 cm^−1^. Multivariant methods in machine or deep learning were finally exploited by the authors to establish regression models for fast automatic analysis (*ca.* 5 min) of acephate levels in rice samples (Fig. 6A). In a recent study, Gao *et al.*^68^ generated core-satellite structures *via* electrostatic assembling of cysteamine-modified gold nanoparticles (core) with negatively-charged mercaptopropionic acid-capped CdTe quantum dots (satellites, QDs), which causes the QD photoluminescence quenching due to energy transfer (Fig. 6B). The addition of the trinitrotoluene (TNT) explosive selectively promotes the detachment of the satellites from the plasmonic core as a result of the formation of the strong cysteamine–TNT complex. Such disassembly is reflected in the TNT concentration dependent changes of the extinction spectra and fluorescent intensity of QDs (Fig. 6C and D), thereby facilitating the naked-eye detection of the explosive. On the other hand, the formation of the cysteamine–TNT complex triggers gold nanoparticle aggregation and the consequent enhancement of the SERS signal (Fig. 6E), which also scales linearly with the target concentration. By combining the trimodal responses, it is possible to achieve an outstanding linear dynamic range of concentration (from 10 fM to 200 μM) with a limit of detection (LOD) of 3.2 fM. The viability of the multichannel sensing platform was validated by TNT detection in environmental and biological specimens such as liquor, fruit, clothing, and soil.

### 3.3 Large biomolecules

**Fig. 7:**
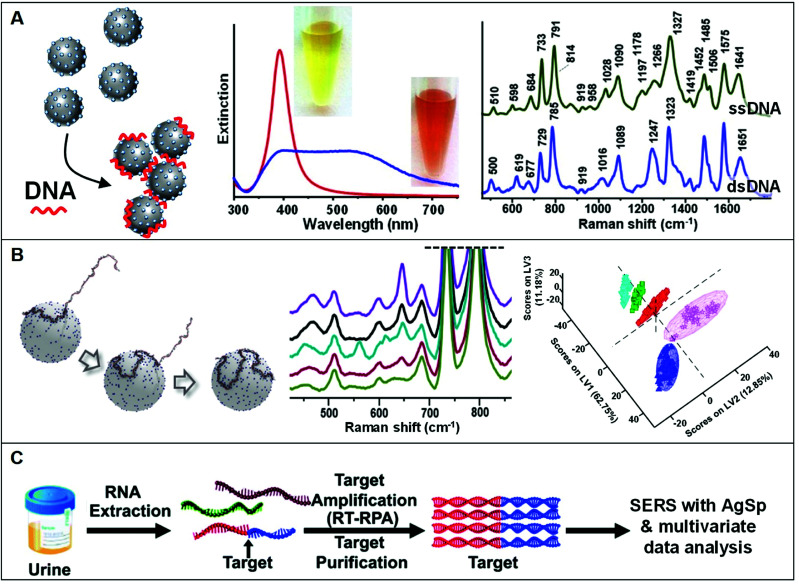
Direct SERS detection of nucleic acids. (A) Schematic of the DNA-mediated assembly of spermine-coated silver colloids (AgSp) into stable clusters in suspension. Adapted with permission from ref. 11 Copyright 2015, Wiley-VCH. Examples of extinction spectra of AgSp before (red curve) and after (blue curve) the addition of a 21-base pair dsDNA (optical images of the colloids are also included). SERS spectra of the ssDNA = CATCGCAGGTACCTGTAAGAG, and the dsDNA consisting of the mentioned ssDNA and its complementary strand. Adapted with permission from ref. 15. Copyright 2021, MDPI. (B) Molecular dynamics simulation of a 141-nt ssDNA adsorption and wrapping around a AgSp nanoparticle at low salt concentration. SERS spectra of 141 nt ssDNAs with single and double-point mutations, and corresponding partial least-squares discriminant analysis (PLS-DA). Adapted with permission from ref. 18 Copyright 2017 WILEY-VCH. (C) Outline of the coupling of a nucleic acid amplification method to direct SERS analysis with AgSp colloids and chemometrics. In this case, total RNA content was initially extracted from urinary samples followed by reverse transcription-recombinase polymerase amplification (RT-RPA) of specific RNA biomarkers to yield ∼200 bp dsDNA amplicons, which were discriminated *via* SERS using spermine-coated silver colloids in combination with multivariate statistical analysis. Adapted with permission from ref. 19 Copyright 2017 Royal Society of Chemistry.

The use of positively-charged nanoparticles as SERS platforms is particularly suited for the analysis of nucleic acids as the negatively-charged phosphate backbone of the biomolecule acts as an electrostatic glue promoting nanoparticle clustering. Such an interaction selectively locates the target sequences at the interparticle gaps (*i.e.*, hot-spots) to maximize their SERS response while simultaneously generating, under optimized conditions, stable clusters in suspension (Fig. 7A). This also avoids the need of using external aggregating agents, as in the case of conventional negatively-charged colloids, which negatively affects the reliability of the SERS outcome mostly due to the poorly controlled and time-dependent colloidal aggregation causing signal fluctuations.^5^ In a seminal work, van Lierop *et al.* prepared silver nanoparticles coated with a wide range of positively-charged ligands (including PLL, PEI, CTAB, spermine, spermidine, and ethylenedioxydiethylamine) and tested their performance in the SERS detection of 5-carboxyfluorescein labeled single-stranded DNA. Among different substrates, spermine-coated nanoparticles (AgSp) allowed the acquisition of much more intense SERS signals with the lowest LOD (1 nM), which has been attributed to, on the one hand, the smaller molecular size as compared to polymers and CTAB layers (*i.e.*, shorter interparticle gaps with larger EM enhancements) and, on the other hand, to the higher affinity for DNA binding as compared to other small amines. On these bases, AgSp were subsequently used as plasmonic substrates in the label-free SERS analysis of nucleic acids, demonstrating their ability to yield very intense and well-defined SERS spectra with an unprecedented level of batch-to-batch reproducibility at the nanogram per mL level. As emphasized in the comparison of the SERS spectra of a single and double-stranded DNA (ssDNA and dsDNA, respectively) reported in Fig. 7A, the obtained spectra provide an accurate and reliable structural fingerprint of the analyzed sample which allows for effective discrimination and classification between nucleic acids (a vibrational band assignment can be found in ref. 5). Our group pioneered this approach^11,18,86–93^ and implemented it in a variety of studies focusing, among others, on the discrimination and quantification of hybridization events and nucleobase modifications,^11,91^ structural classification of diverse classes of RNAs,^88^ quantification of nucleobase content,^89^ characterization of DNA interaction with exogenous molecules,^92^*etc.* In particular, we demonstrated the viability of this method for being integrated into clinical applications differentiating clinically relevant point mutations in 141-nt K-Ras oncogene segments (Fig. 7B).^18^ In combination with partial least-squares discriminant analysis (PLS-DA), a well-established statistical classification method, we were able to discriminate diverse patterns for SERS spectra of large ssDNA strand with single-point mutations. Differently to short sequences which linearly extends over the metallic surface, AgSp nanoparticles function as compaction agents for long strands stimulating their folding into different structural forms (A- and B-forms, and a combination of thereof) depending on the specific nucleobase sequence and composition, as consistently suggested by the spectral changes of diagnostic backbone conformation bands. This study was conducted in buffered solutions while, in clinical samples (*e.g.*, blood, urine, saliva), nucleic acids typically coexist, at very low concentrations, with a complex mixture of other molecules that compete for adsorption onto the plasmonic surface. For this reason, upstream amplification processes (*e.g.*, polymerase chain reaction, PCR) are necessary to selectively enrich the target sequences while reducing their lengths and separate it from the biological media.^19,57,60,94^ Trau and co-workers implemented such an approach by utilizing multiplex reverse transcription-recombinase polymerase amplification (RT-RPA) to enrich prostate cancer (PCa) RNA biomarkers (T1E4 and RN7SL1) from urine samples.^19^ As outlined in Fig. 7C, the target RNA sequences are amplified as *ca.* 200 bp dsDNA amplicons, which are separately interrogated by SERS using AgSp colloids. Multivariate statistical analysis of the data allowed the risk stratification of PCa in 43 patient urinary samples with high specificity, sensitivity, and accuracy. In a second study,^57^ the authors successfully extended such a method beyond the proof-of-concept stage to validate the clinical performance of the nanodiagnostic tool by evaluating the clinical metrics with a well-suited cancer risk stratification scoring model. Similarly, Liu *et al.*^60^ implemented polymerase chain reaction (PCR), direct SERS interrogation and principal component analysis-linear discriminant analysis (PCA-LDA) to identify and classify BRAF wild type (WT) and V600E mutant genes, firstly, from both genome DNA (gDNA) and cell-free DNA (cfDNA) collected from cell culture media and, subsequently in plasma samples. In this case, however, the authors tested the SERS enhancing performances of different spermine-coated plasmonic nanoparticles (*i.e.*, including gold or silver nanospheres, nanoshells, nanoflowers, and nanostars). Under the reported experimental conditions, gold/silver nanostars showed the highest SERS activity for DNA detection.

In addition to nucleic acids, Liu *et al.*^53^ also employed AgSp colloids for monitoring protein kinase A (PKA) activity in cell extracts. PKA-induced phosphorylation selectively alters the net charge state of a dye-labelled peptide (TAMRA-LRRASLG) which promotes the electrostatic interaction with silver nanoparticles and their aggregation. The intensity of the SERS spectra of the TAMRA dye quantitatively correlates with PKA concentration in a wide range of 0.0001–0.5 U μL^−1^ with a LOD equals to 0.00003 U μL^−1^.

It is also worth noting that the structural and functional plasticity of dsDNA to simultaneously yield stable clusters of AgSp nanoparticles in suspension and non-covalently interacting with small aromatic molecules *via* intercalative binding was exploited in the simultaneous quantification of Fe(iii) and Al(iii) in tap water.^95^ Specifically, in this study, dsDNA complexation with the anthraquinone derivative alizarin red S (ARS) prevents the direct interaction of ARS with the plasmonic surface while locating it close enough to the silver surface to yield intense SERS signals. In this scenario, the ARS chelating properties towards iron and aluminum ions are retained and well-revealed in large changes in the SERS spectral profiles, which can be quantitatively correlated with the ion concentration. In the absence of dsDNA, ARS molecules directly adhere onto the AgSp surfaces through the same coordination sites for metal ion binding, thereby hindering their use as SERS chemosensors.

### 3.4 Pathogens and vesicles

**Fig. 8:**
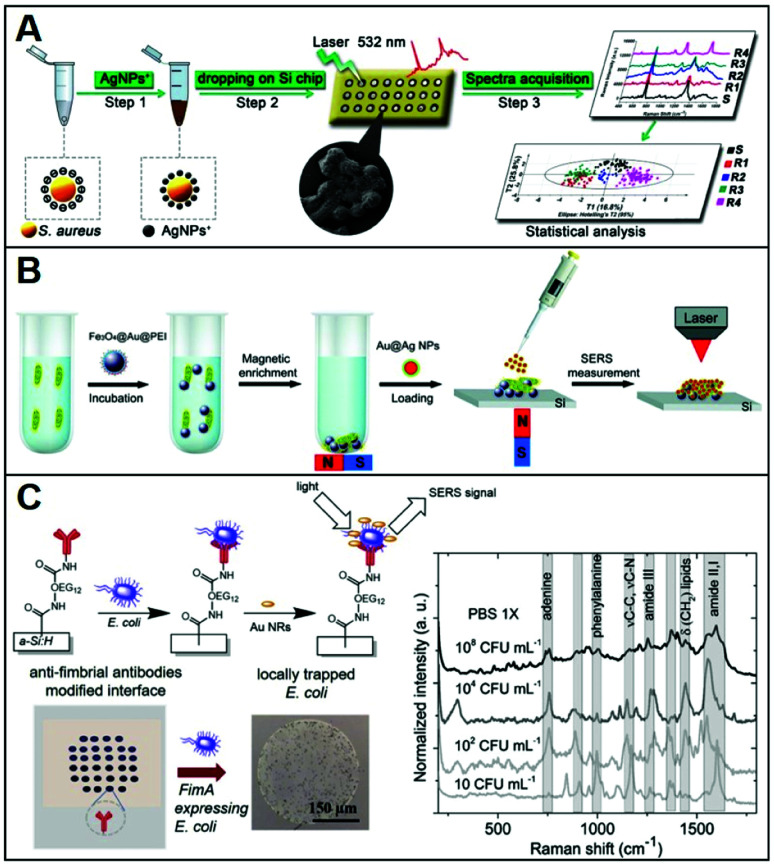
Direct SERS identification of pathogens. (A) Schematic of the direct SERS analysis of pathogens using positively-charged plasmonic nanoparticles which involves the electrostatic accumulation of the nanoparticles at the cell membrane, the deposition of the sample onto a solid support, and subsequent SERS interrogation. Chemometric methods are used for data analysis. Adapted with permission from ref. 51 Copyright 2019, Springer. (B) Outline of the magnetic/SERS sensing strategy for bacteria detection. Step 1: Fe_3_O_4_@Au@PEI are used to capture and concentrate pathogens from the media *via* electrostatic interactions. Step 2: acquisition of the pathogen SERS spectra *via* deposition of a compact layer of citrate-capped Au@Ag nanoparticles on dried Fe_3_O_4_@Au@PEI–bacteria samples. Adapted with permission from ref. 75 Copyright 2016, Royal Society of Chemistry. (C) Bacteria selective capturing at hydrogenated amorphous silicon surfaces modified with anti-fimbrial antibodies and SERS detection *via* electrostatic adhesion of CTAB-caped gold nanorods. The illustration also includes optical images of the anti-fimbriae modified array and the spots after interaction with bacteria. Averaged and normalized SERS spectra of captured *E. coli* K12 MG1655 extracted from SERS mapping of the trapped bacteria at different *E. coli* concentrations (10^8^, 10^4^, 10^2^ and 10 CFU mL^−1^) in PBS 1×. Adapted with permission from ref. 46 Copyright 2020, Elsevier.

Conventional methods for microorganism detection (*e.g.*, culture-based approaches) are laborious and very time-consuming, while other molecular diagnostic tools (*e.g.*, ELISA, PCR) suffer from high cost and/or low sensibility. Thus, SERS has been explored as an analytical tool for the rapid, low-cost, and sensitive detection of pathogens (*e.g.*; bacteria, fungi), adopting diverse indirect^96,97^ and direct sensing strategies, along with positively-charged nanoparticles.^27,34,38–40,46,47,50,51,75^ A representative workflow to carry out direct SERS analysis of pathogens using AgNPs is outlined in Fig. 8A. In this work, Chen *et al.*^51^ use CTAB-coated silver nanoparticles for the identification of methicillin-resistant *Staphylococcus aureus* (MRSA) as well as discrimination of 6 microorganisms (*S. aureus* 29213, *S. aureus* 25923, *C. albicans*, *B. cereus*, *E. coli*, and *P. aeruginosa*). A pathogen suspension prepared with ultrapure water was incubated for 30 min with positively charged nanoparticles, which self-assemble on the microorganism membrane *via* electrostatic adhesion (conversely, citrate-capped silver nanoparticles poorly accumulate at the pathogen surface). Samples were then dried on a silica chip to be investigated by SERS, yielding the corresponding spectra which contain an ensemble of features ascribed proteins, lipids and DNA, mostly arising from membrane components. Due to the spectral similarity between pathogens, chemometric methods (*e.g.*, partial least squares discriminant analysis, PLS-DA) are appropriately implemented for data processing and accurate spectroscopic discrimination of different microorganisms. Adopting a similar sensing scheme, two different species of *Cryptococcus* (*Cryptococcus neoformans* and *Cryptococcus gattii*) with different pathogenicity, diagnosis, and treatment were accurately discriminated using CTAB-coated silver nanoparticles and direct SERS analysis in combination with orthogonal partial least-squares discriminant analysis.^47^ In another study, discrimination of three bacteria (*Escherichia coli, Salmonella typhimurium*, and *Bacillus subtilis*) was achieved with excellent reproducibility by using PEI-coated bimetallic Ag/Au nanoparticles. Here, bacteria were drop-cast onto a slide and then exposed to the nanoparticles to yield intense SERS signals.^39^ Interestingly, chitosan-coated silver nanoparticles mixed with two strains of Gram-positive *Staphylococcus aureus* have been shown to exhibit a synergistic antibacterial activity.^38^ Direct SERS analysis is then used to monitor the resulting biochemical changes in the bacterial cell wall.

Magnetic functionalities were also integrated into the SERS sensing schemes to facilitate the separation and accumulation of the pathogens at a specific point of the slide while removing the need for time-consuming and complex procedures.^50,75^ Notably, amine-functionalized magnetic particles have shown to be used as effective capture agents for microrganisms such as bacteria over a wide pH range (5.0–8.0).^98^ For instance, PEI-coated magnetic particles (Fe_3_O_4_@PEI) were used to electrostatically capture *Candida* species causing fungal infections (*Candida albicans, Candida tropicalis*, and *Candida krusei*) from serum with high efficiency (step 1) and, once separated from the media, mixed with CTAB-coated silver nanoparticles and, finally, deposited on a silicon wafer for SERS analysis (step 2).^50^ Multivariate data analysis was used to accurately discriminate among the *Candida* species; the whole test can be completed within 40 minutes.

Alternatively, Wang *et al.*^75^ afforded plasmonic properties to the magnetic microbeads by integrating an external layer of gold nanoparticles prior to the final PEI-coating (*i.e.*, Fe_3_O_4_@Au@PEI). These substrates simultaneously allow for the rapid separation and accumulation of bacteria from the media and their SERS detection (Fig. 8B). However, the obtained LODs were relatively high (*ca.* 1 × 10^5^ cells per mL for *E. coli*) thus an additional step was integrated to enhance the sensitivity of the platform. Specifically, a concentrated suspension of citrate-capped Au@Ag nanoparticles dissolved in ethanol was deposited on the dried sample (*i.e.*, Fe_3_O_4_@Au@PEI–*E. coli* complexes). The increased Au@Ag nanoparticles content and the use of ethanol as a solvent ease the formation of a densely packed layer of nanoparticles around the beads thanks to the rapid evaporation of the solvent. With such an increased density of hot-spots around the captured bacteria, Gram-positive bacterium *E. coli* and Gram-positive bacterium *S. aureus* can be detected at LOD as low as 10^3^ cells per mL. Validity of the method was also demonstrated in tap water and milk spiked with bacteria, while the optimization of the experimental conditions allowed achieving rapid bacterial detection within 10 minutes (principal component analysis, PCA, was applied in this work).

Otherwise, for selective capturing of a specific class of bacteria from biological samples, appropriate antibodies, when available, can be used. In this regard, Andrei *et al.*^46^ fabricated a biochip consisting of a glass substrate coated with a thin film of hydrogenated amorphous silicon, which has been conjugated with anti-fimbrial *E. coli* antibodies for the specific trapping of *E. coli* strains containing type-1 fimbriae. The capturing surface was also modified with CH_3_O–PEG750, an oligoethylene glycol molecule that imparts anti-fouling character (Fig. 8C), preventing unspecific adhesion. In a fluid cell, the bacteria containing suspension is put into contact with the biochip surface through cycles of flow and static contact stages to maximize bacteria trapping. Subsequently, CTAB-capped gold nanorods are deposited on the support and a SERS mapping is performed on the dried sample. The bacterial vibrational fingerprint of *E. coli* is preserved in the biochip configuration. As shown in the SERS spectra in Fig. 8C, characteristic features ascribed to amide bands, phenylalanine stretching deformation of lipidic C–H bonds, and adenine vibrations are clearly discernible. The applicability of the method was demonstrated in artificial urine spiked with *E. coli* K12, where reproducible SERS fingerprints of the bacteria have been acquired down to 10 colony-forming units (CFU) per mL, far below the clinical threshold, in less than 3 h.

The electrostatic interactions of positively-charged nanostructures and negatively-charged biological membranes have been also exploited in the direct SERS analysis of exosomes in combination with multivariate analysis.^2,59,99–101^ Exosomes are heterogeneous single-membrane vesicles with diameters between 30 and 150 nm and secreted by almost all cells, which are emerging as one of the most compelling biomarkers in liquid biopsies for early cancer diagnosis, prognosis, and treatment monitoring.^2^ Among others, Shin *et al.*^99^ functionalized a compact film of gold nanoparticles with cysteamine to favor the accumulation of exosome suspension at the SERS mapping areas. In addition to the spectral differentiation between normal (HPAEC) and lung cancer (H1299, PC9) cell lines, the molecular origin of the Raman markers at the basis of such discrimination was elucidated, indicating the membrane protein EGFR as the primary source of exosomal differentiation. Analogously, Rojalin *et al.*^101^ used cysteamine to impart a positive charge on silver nanoparticles integrated onto microscale biosilicate materials for SERS analysis of ovarian and endometrial cancer exosomes. On the other, Liu *et al.*^59^ employed spermine-coated gold nanostars to acquire the SERS fingerprint of vesicles from different isolation protocols, batches, and cell origins, demonstrating the viability of such an approach to correctly classify the vesicles obtained using three different isolation methods.

### 3.5 Cell SERS imaging

**Fig. 9:**
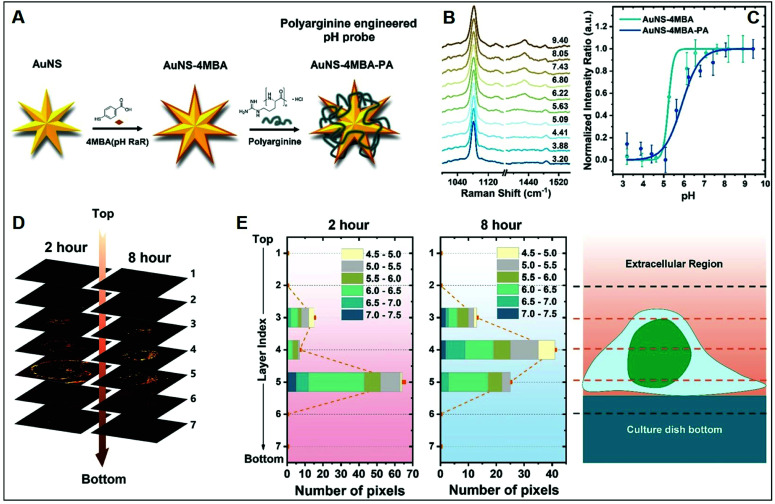
Cell SERS imaging. (A) Fabrication scheme of the preparation of pH-sensitive plasmonic sensor comprising gold nanostars (AuNS) functionalized with 4MBA (4-mercaptobenzoic acid) as the pH-sensitive molecular reporter and wrapped with polyarginine. (B) pH-dependent SERS spectra of the probes in complete cell culture media. (C) SERS intensity ratios (*I*_1425_ + *I*_1429_)/*I*_1081_*vs.* pH of the cell culture media for MBA-functionalized AuNSt with and without PA coating. (D) Reconstruction of 3D SERS imaging of the internalization of the nanoparticles into a single MCF7 cell at different *x*–*y* planes separated by 2 μm along the orthogonal *z*-axis upon 2 and 8 hours incubation. The brightest areas correspond to larger nanoparticle accumulations. (E) pH range distribution at the different *x*–*y* planes as a function of pixel number. Adapted with permission from ref. 26 Copyright, American Chemical Society, 2020.

The molecular specificity, sensitivity, and high special resolution of SERS are particularly suited for living cell imaging and the dynamic monitoring of biological processes at the cellular and subcellular level through the use of appropriately designed biocompatible plasmonic nanoparticles.^102^ To this end, nanoparticle surface properties are, among others, tuned to afford long-term stability in cell media and efficient cellular internalization, a feature that is strictly related to nanomaterial surface charge.^24,26^ As a representative example, we will discuss the work performed by Liz-Marzan and co-workers,^26^ in which high-resolution 3D SERS imaging of the intracellular pH was performed in living MCF7 breast cancer cells. The pH-responsive plasmonic nanosensor comprised gold nanostars (AuNSt) functionalized with 4-mercaptobenzonic acid (4-MBA) as a pH-sensitive molecule and subsequently wrapped with an external layer of the cationic poly-l-arginine hydrochloride (PA) polymer *via* electrostatic interactions (Fig. 9A). The cationic polymer shell equips the pH-SERS sensitive probes with high colloidal stability and biocompatibility as well as a higher degree of cellular internalization as compared to other classes of protective layers yielding neutral or negative charge due to the ability of PA to adhere onto the cell membrane *via* electrostatic interactions and facilitate endocytosis and translocation in cells.^103^Fig. 9B shows the pH-dependent MBA SERS profiles of the nanosensors in cell culture media. Most notably, protonation and deprotonation of the carboxylic group make COO^−^ stretching modes at 1415/1430 cm^−1^ particularly sensitive to changes in pH, contrarily to the intense 1081 cm^−1^ feature, mainly associated with ring breathing of the phenyl moiety. Thus, the intensity ratio between the sum of the 1415 and 1429 cm^−1^ contributions and the 1081 cm^−1^ band was chosen as the spectral marker of the proton concentration and plotted in Fig. 9C as a function of pH. Interestingly, the PA coating also improves the quality of the pH response curve (*i.e.*, milder slope in the 4–8 pH range), which has been tentatively ascribed to the effect of the PA permeability on the diffusion of ions as well as the structural impact that the PA layer may have on the underlying 4MBA molecules. Then, MCF7 cells were exposed to the nanoparticles for 2 or 8 hours and 3D imaging was carried out at 2 μm separated *x*–*y* planes along the orthogonal *z*-axis. Fig. 9D shows the corresponding stacked 2D images of SERS intensities which localize the 3D distribution of the nanoparticles at the levels 3–5, in agreement with the volumetric height of a live MCF7 cell (*ca.* 6–8 μm). Nanoparticle cellular distribution is visualized in Fig. 9D by plotting the number of pixels with distinguishable SERS intensity at 1081 cm^−1^ at the different *x*–*y* planes. Furthermore, the pH value is also extracted from the SERS spectra as previously discussed. Direct comparison of results at *t* = 2 h and 8 h suggests a progressive motion of the nanoparticles from the cell membrane toward the perinuclear area which is accompanied by a drop of the environmental pH from 6–7 during the first stage of cellular endocytosis (endosomal compartment) to 4.5–5, when nanoparticles are entrapped into mature lysosomes.^104^

On the other hand, Sujai *et al.*^24^ showed that, while imparting a positive charge to nanoparticles enhances their cellular internalization, it depresses their ability to penetrate within multilayered cell assemblies. In this work, the authors synthesized SERS-encoded gold nanoparticles coated either with thiolated-polyethylene glycol (HS-PEG) or polyallylamine (PAA) to afford colloidally stable negative and positive materials, respectively. Uptake efficiency was monitored *via* SERS in monolayer (2D) cells and multi-layered spheroids (3D), as a model system for living tissues. Positively-charged NPs demonstrated their faster internalization *via* an endocytic pathway in monolayer cells but *ca.* a 9 times lower penetration capability in cell spheroids as compared to negatively-charged SERS-encoded NPs. This has been attributed to the electrostatic repulsion between negatively-charged cell membranes (and other biological macromolecules) and negatively charged SERS-encoded NPs, which would propel the diffusion of the particles through the interstitial spaces and, eventually, their internalization into deep-seated cells. The results clearly highlight the importance of the rational design of the nanomaterials to be used as efficient nanotheranostic agents. In fact, positively-charged NPs appear to be the optimal candidates for rapid uptake by small sized tumors, while negatively-charged NPs are expected to penetrate deeper into the core of larger tumors.

## 4. Conclusions

In this review, we summarized and discuss the main procedures to prepare positively-charged plasmonic nanomaterials *via* surface functionalization with amine ligands of very different nature and size, using different synthetic strategies. Successively, a comprehensive list of studies integrating such substrates for SERS sensing was also examined and liberally classified according to the size of the target analyte which structural properties outline, by and large, the class of nanomaterial and the respective type of amino surface ligands that are more suited for the specific purpose. Future challenges regarding the engineering and exploitation of this sub-class of nanomaterials largely overlap those of all substrates designed for analytical SERS applications, most notably (i) the affordable production of reliable, robust, and effective plasmonic platforms obtained *via*, ideally, simple standardized protocols, and (ii) the integration into multifunctional systems (*e.g.*, microfluidics, magnetic, multi-optical read-outs for enhanced sensing, chemometrics, *etc.*) to overcome the intrinsic limitations of SERS as stand-alone technique and enable automatization/standardization of the measuring procedures. On a more specific note, we emphasize a certain technological delay in the development and design of positively-charged nanomaterials as compared to the state-of-the-art nanofabrication research, which it may be ascribed to the more recent implementation of this sub-class of nanostructures to relevant applications. Similarly, mono and polyamine molecules exploited as surface ligands to fabricate positively-charged nanomaterials have been selected so far only among those commercially available. As demonstrated, even minor structural changes in the amine ligands can play a central role at determining the SERS analytical performances of the material. Thus, we expect that à *la carte* synthesis of different amine molecules, such as thiolated-spermine analogs, could significantly improve the quality of the sensing platform, also in terms of robustness and reusability, with particular focus on paving the way for their application in complex biological media for SERS bio-sensing.

## Conflicts of interest

There are no conflicts to declare.

## Supplementary Material
